# Genome-wide characterization of regulator of chromosome condensation 1 (*RCC1*) gene family in *Artemisia annua* L. revealed a conservation evolutionary pattern

**DOI:** 10.1186/s12864-023-09786-4

**Published:** 2023-11-18

**Authors:** Jieting Chen, Wenguang Wu, Xiaoxia Ding, Danchun Zhang, Chunyan Dai, Hengyu Pan, Peiqi Shi, Chanjuan Wu, Jun Zhang, Jianmin Zhao, Baosheng Liao, Xiaohui Qiu, Zhihai Huang

**Affiliations:** 1 Key Laboratory of Quality Evaluation of Chinese Medicine of the Guangdong Provincial Medical Products Administration, the Second Clinical College, Guangzhou University of Chinese Medicine, Guangzhou, 510006 China; 2 Artemisinin Research Center, China Academy of Chinese Medical Sciences, Beijing, 100700 China; 3 Sunribio Co.Ltd, Shenzhen, 518101 China

**Keywords:** *Artemisia annua*, *RCC1*, UVR8, UV-B, Purifying selection

## Abstract

**Background:**

*Artemisia annua* is the major source for artemisinin production. The artemisinin content in *A. annua* is affected by different types of light especially the UV light. *UVR8*, a member of *RCC1* gene family was found to be the UV-B receptor in plants. The gene structures, evolutionary history and expression profile of *UVR8* or *RCC1* genes remain undiscovered in *A. annua*.

**Results:**

Twenty-two *RCC1* genes (*AaRCC1*) were identified in each haplotype genome of two diploid strains of *A. annua,* LQ-9 and HAN1*.* Varied gene structures and sequences among paralogs were observed. The divergence of most *RCC1* genes occurred at 46.7 – 51 MYA which overlapped with species divergence of core Asteraceae during the Eocene, while no recent novel *RCC1* members were found in *A. annua* genome. The number of *RCC1* genes remained stable among eudicots and *RCC1* genes underwent purifying selection. The expression profile of *AaRCC1* is analogous to that of *Arabidopsis thaliana (AtRCC1)* when responding to environmental stress.

**Conclusions:**

This study provided a comprehensive characterization of the *AaRCC1* gene family and suggested that *RCC1* genes were conserved in gene number, structures, constitution of amino acids and expression profiles among eudicots.

**Supplementary Information:**

The online version contains supplementary material available at 10.1186/s12864-023-09786-4.

## Background

*Artemisia annua*, a traditional Chinese medicine, belonging to Asteraceae family, is the major source for artemisinin which is widely used in the treatment of malaria [1, 2]. Artemisinin-based combination therapies (ACTs) have been highly recommended by the World Health Organization for treating malaria [3–5]. Though semisynthetic artemisinin has been developed [6, 7], low yield and high cost make large-scale industrial applications unavailable [8]. Currently, *A. annua* is the major source of artemisinin.

The main distribution areas of *A. annua* were concentrated in mid-latitudes in southeastern Asia, western and central Europe, south-eastern North America and south-eastern South America [9]. In China, *A. annua* grown in the south of the Qinling Mountains-Huaihe River Line had a higher artemisinin content compared to the northern ones [10]. Humidity and sunshine duration were speculated as major limiting ecological factors that affect the accumulation of artemisinin [11]. *A. annua* is a determinate short-day plant with a critical photoperiod [12], while biomass and artemisinin production were increased in response to long-day photoperiod [13]. The daylight contains a variety of radiation, of which the Ultraviolet-B radiation (UV-B, 280–315 nm) [14] is an important environmental signal that pleiotropically regulates development, morphogenesis and physiology in plants [15]. Previous studies have demonstrated that short-term UV-B treatment to *A. annua* may be a safe approach to accumulating artemisinin content while acting on stress-regulated genes to keep the plant healthy [16]. Besides, UV-B radiation and phytohormone gibberellins coordinately promoted the accumulation of artemisinin in *A. annua*, with a significant up-regulation of two genes in artemisinin biosynthetic pathway (*ADS* and *CYP71AV1*) [17].

UV RESISTANCE LOCUS 8 (UVR8) is an evolutionarily well conserved UV-B photoreceptor that regulates UV-B photomorphogenesis in plants [18], which employs a unique photosensory mechanism for light absorption and initiation of the signaling events that eventually lead to particular physiological responses [19–21]. UVR8 contains sequence similarity and predicted structural similarity to human Regulator of Chromatin Condensation 1 (RCC1), whose sequence is highly conserved among all eukaryotes and consists of a seven-bladed-β-propeller, also known as seven RCC1 repeat units [22, 23]. *RCC1* functions as a guanine-nucleotide-exchange factor (GEF) for the Ran G-protein to regulate diverse biological processes, nucleocytoplasmic transport, and the cell cycle [24]. *UVR8* is a member of *RCC1* gene family, which strongly associates with chromatin, while UVR8 has little Ran GEF activity and it is present in both the cytosol and nucleus in contrast to other *RCC1* family proteins localized in the nucleus [25]. Normally, UVR8 is evenly distributed in the cytoplasm and nucleus, however, under UV-B treatment, it tends to accumulate in the nucleus through interaction with constitutive photomorphogenic 1 protein (COP1), triggering a UV-B cascade [18, 26, 27]. The amino acid sequence of UVR8 is enriched with aromatic residues [28]. The aromatic amino acids refer to amino acids with benzene ring in molecular structure, including tyrosine (F), phenylalanine (P) and tryptophan (W), which is bound up with UV absorption [29]. The *Arabidopsis thaliana* (AtUVR8) has 14 W residues, among which W285 and W233 were shown to have an important role in UV-B-triggered signaling [30, 31]. The participation of UVR8 in the UV-B response is UV-B dose-dependent, which mediates several responses to low doses of UV-B, while high UV-B doses trigger other adaptive mechanisms [32]. The UVR8 and other members in the *RCC1* gene family have been identified in a range of plant species [33, 34]. For instance, *Spartina alterniflora* RCC1 (SaRCC1), negatively regulates salt stress responses by affecting stress-related gene expression [33]. RUG3 (a mitochondrial protein) is required for efficient splicing of the nad2 mRNA, which encodes a complex I subunit in mitochondria of *A. thaliana* [35]. Tolerant to Chilling and Freezing 1 protein (TCF1), interacts with histones H3 and H4 and associates with chromatin containing a target gene, encoding a glycosylphosphatidylinositol-anchored protein that regulates lignin biosynthesis, and thus affect the freezing tolerance of plants [36, 37]. RCC1-like domain (RLD) proteins, identified as LZY interactors, are essential regulators of polar auxin transport [38]. SAB1 is a crucial new component of ABA signaling which negatively regulates ABI5 through multidimensional mechanisms during post-germination in *A. thaliana* [39]. Currently, the *UVR8* and *RCC1* gene family in *A. annua* genome has not been reported as well as their evolutionary history.

In this study, a comprehensive bioinformatic analysis was conducted on the *RCC1* gene family at the genome-wide level of four haplotype genomes of two *A. annua* strains, including gene structures, phylogenetic relationship construction, gene variation and gene expression profile, which could provide useful information for further functional investigations of *A. annua*.

## Results

### Identification and characterization of *AaRCC1* genes

**Fig. 1:**
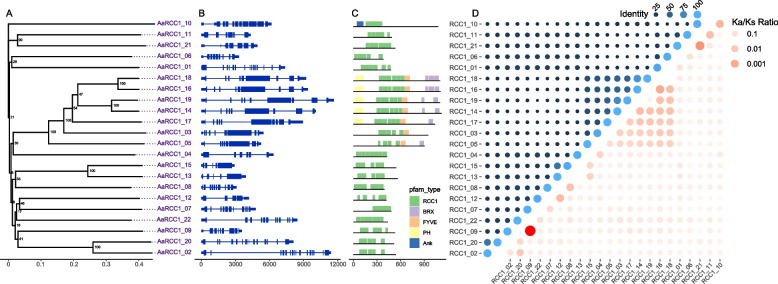
Characteristics of *AaRCC1* genes. **A** Phylogenetic relationships (numbers on the nodes represent supporting values). **B** Gene structures. Blue rectangles represent the coding sequences, thin blue lines connecting two exons represent introns, and thick blue lines represent 5′-UTR or 3′-UTR. **C** Domain information identified by PfamScan. **D** Identity and Ka/Ks values between alleles

Genes with *RCC1* domain (PF00415) were defined as candidate *RCC1* genes and then manually corrected. In total, 22 *RCC1* genes (named *AaRCC1_01* to *AaRCC1_22*) were identified in each haplotype of *A. annua* (LQ-9 haplotype 0 and haplotype 1, HAN1 haplotype 0 and haplotype 1) (Fig. 1A and Table S1). *RCC1* gene number was consistent among four haplotypes. Gene structures varied among gene members (Fig. 1B). The exon numbers varied from 4 to 16, and gene length ranged from 2, 911 (*AaRCC1_15*) to 11, 666 (*AaRCC1_19*) bp (Table 1). The RCC1 domain number varied from 4 to 7 and some members had PH (PF00169), BRX (PF08381) or FYVE (PF01363) domain (Fig. 1C). Genes with the same exon number and function domain annotated in *RCC1_14*, *16*, *17*, *18*, *19* clustered in a same clade, showing similarities on gene structures and domain regions. The average content of tryptophan (1.96%) and aromatic amino acids (7.54%) in AaRCC1 proteins were significantly higher than those of other proteins in the whole genome (average tryptophan 1.38%, average aromatic amino acids 3.19%, p-value < 0.05). The AaRCC1_22 showing the highest protein identity (76.52%) to that of *Arabidopsis thaliana* (AtUVR8) was identified as AaUVR8, which has relatively high W content in protein sequences among all *RCC1* genes (Table 1). All CDS and protein sequences of *RCC1* genes were aligned pairwisely. High similarities were detected among alleles of each *AaRCC1* gene (CDS sequence identity 79.61–100%, protein sequence identity 94.20%-100%), while sequence variation existed (protein sequence identity 10.43%-82.11%) (Fig. 1D). Notably, most of the Ka/Ks values calculated between alleles and gene members were less than 1, indicating these *RCC1* genes were under purifying selection (Fig. 1D) and tend to eliminate deleterious mutations and maintain functional stability [40].

**Table 1:** The basic information about the *RCC1* genes in *A. annua* LQ-9 haplotype 0

Gene name	Gene length (bp)	CDS length (bp)	Intron/ Exon	Pep length	MW	PI	Tryptophan content (%)	Aromatic amino acid content (%)
*AaRCC1_01*	7375	1434	14/15	477	51,568.61	6.5	1.89	7.06
*AaRCC1_02*	11,400	1624	15/16	540	58,375.81	9.37	1.48	9.01
*AaRCC1_03*	5477	2853	7/8	950	104,348.87	6.92	1.26	6.11
*AaRCC1_04*	6354	1287	5/6	428	45,206.27	6.64	2.57	7.68
*AaRCC1_05*	5265	2718	7/8	905	99,733.53	9.19	1.55	7.24
*AaRCC1_06*	3312	1149	8/9	382	40,711.91	5.6	2.36	7.18
*AaRCC1_07*	4776	1449	10/11	482	50,596.17	5.62	2.90	6.54
*AaRCC1_08*	3089	1188	9/10	395	42,267.78	5.85	3.80	7.26
*AaRCC1_09*	3568	1593	8/8	466	50,337.21	6.15	2.58	6.58
*AaRCC1_10*	6180	3240	10/10	1079	117,690.82	9.36	1.39	8.80
*AaRCC1_11*	4340	1470	5/6	489	53,398.99	7.53	1.02	5.75
*AaRCC1_12*	4192	1269	4/4	422	44,822.19	5.37	2.84	9.41
*AaRCC1_13*	3921	1695	4/5	536	58,024.84	5.43	2.80	6.16
*AaRCC1_14*	10,082	3366	8/9	1121	121,840.35	8.65	1.34	8.58
*AaRCC1_15*	2911	1638	4/5	545	58,757.24	5.12	2.94	6.42
*AaRCC1_16*	9373	3321	8/9	1106	119,713.11	9.08	1.45	8.99
*AaRCC1_17*	8934	3117	9/10	1038	114,125.44	8.83	1.25	6.33
*AaRCC1_18*	9223	3306	8/9	1101	119,920.67	9.11	1.45	7.03
*AaRCC1_19*	11,666	3309	8/9	1102	120,245.48	8.89	1.45	6.18
*AaRCC1_20*	8104	1554	14/15	517	54,888.78	7.57	1.55	6.90
*AaRCC1_21*	4945	1559	6/5	532	57,086.69	5.91	1.13	6.00
*AaRCC1_22* (*AaUVR8*)	8141	1320	11/12	439	47,637.46	5.44	2.96	7.33

## *RCC1* genes are conserved during speciation in eudicot

**Fig. 2:**
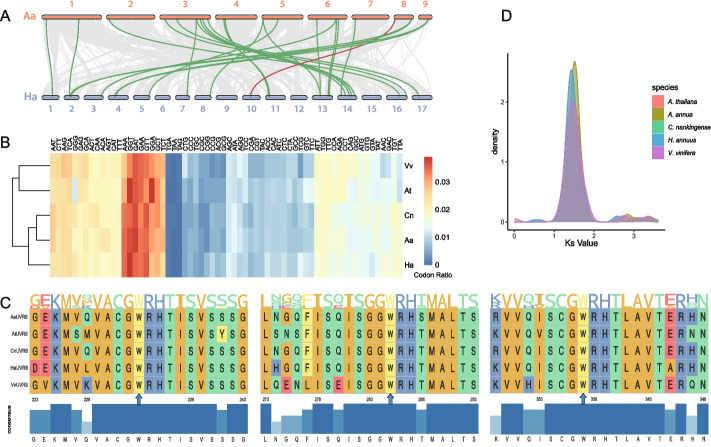
The feature of RCC1 family in five species. **A** The syntenic relationship of *A. annua* and *H. annuus*, green lines represent syntenic *RCC1* gene pairs (LQ-9 haplotype 0), the red line indicates  the *UVR8* gene pair, while the grey background represents other syntenic gene pairs. **B** The codon usage ratio of *RCC1* orthologs in five species. **C** The multiple protein sequence alignment of UVR8 in 5 species. **D** The distribution of *Ks* value of *RCC1* family members in five species

A comparison analysis of *RCC1* genes between *A. annua* and four other species was conducted. We found a similar gene number of *RCC1* family in five species. There are 24 *RCC1* members in *A. thaliana*, 22 in *A. annua*, 27 in *H. annuus* (Fig. 2A), 23 in *Chrysanthemum nankingense* and 21 in *Vitis vinifera*. In contrast with gene families like terpene synthase (*TPS*) [41] and UDP-glucuronosyltransferase (*UGT*) [42, 43], the gene number of *RCC1* remained conserved without significant expansion by segmental/tandem duplication or whole genome polyploidization. However, duplication debris of *RCC1* was identified for *AaRCC1_07* and *AaUVR8* in *A. annua* genome and duplicated genes were functionally silenced by corrupting of gene structures (Figure S2). Furthermore, similar codon usage was found among *RCC1* orthologs (Fig. 2B). Protein sequences were conserved among *RCC1* orthologs across the five species. For example, the UVR8 showed high conservation on protein sequences of AtUVR8 (*A. thaliana*), CnUVR8 (*C. nankingense*), HaUVR8 (*H. annuus*), AaUVR8 (*A. annua*) and VvUVR8 (*V. vinifera*), especially on W233, W285 and W337 that related to UV-B response functions [20, 44] (Fig. 2C). Ks values among orthologs in each species were calculated pairwisely, which were enriched at a peak near Ks = 1.46 (Fig. 2D). The *RCC1* genes were diverged at 46.7 to 51 MYA based on Ks and r value from *A. thaliana* [45], which overlapped with the time of most subfamilies of core Asteraceae diverged during Eocene [46]. Few novel *RCC1* genes were identified after Eocene.

### *AaRCC1* exhibited tissue and treatment specific expression profile

**Fig. 3:**
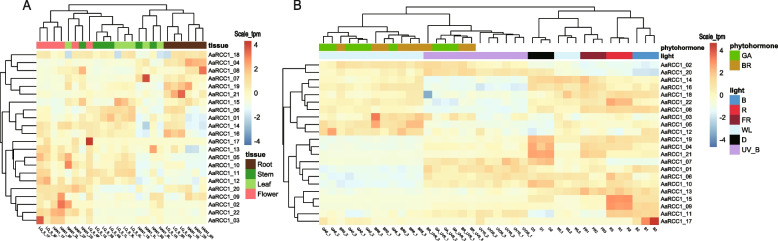
The expression patterns of *AaRCC1* genes. **A** The expression profile of 22 *AaRCC1* genes in four tissues of two strains. **B** The expression profile of 22 *AaRCC1* genes under different treatments. GA, gibberellin; BR, brassinolide; B, blue light; R, red light; FR, far red light; WL, white light; D, dark; UV_B, UV-B radiation

The expression profile of *AaRCC1* genes was examined in different tissues (root, stem, leaf, flower) and different treatments (lights with different wavelengths, including UV-B, blue, red, far-red and white light, phytohormones including gibberellin and brassinolide). The results demonstrated that 22 *RCC1* genes exhibited distinct expression patterns among various conditions (Fig. 3). The expression level of *AaUVR8* in flower was significantly higher than that of other three tissues in two *A. annua* strains (2.4 to 2.7 fold change compared to other tissues, p-value < 0.05). The expression level of *AaRCC1_05* was significantly down-regulated in roots (4.4 to 1.3 fold change compared to other tissues, p-value < 0.05). *AaRCC1_15* and *AaRCC1_17* had higher expression levels in leaves of LQ-9 than those of HAN1. Instead, *AaRCC1_08*, *AaRCC1_16, AaRCC1_04, AaUVR8 and AaRCC1_07* had higher expression levels in leaves of HAN1, which indicated *RCC1* genes of different strains also showed different expression levels. Expression quantification by qRT-PCR of *AaUVR8* showed 1 to 2.6 fold changes among 18 different strains (Figure S3). Interestingly, after UV-B treatment, five *RCC1* genes including *AaUVR8* showed a decreased gene expression, while *AaUVR8* up-regulated significantly with red light treatment. In contrast, two negative response genes, REPRESSOR OF UV-B PHOTOMORPHOGENESIS 1 (RUP1) and RUP2 [47] were up-regulated after UV-B treatment. A similar expression pattern was detected in *A. thaliana* (Figure S4).

## Discussion

*RCC1* genes were found to be regulating factors for a series of downstream genes during biological processes, including stress responses under abiotic stress and various hormone treatments [33–39]. Twenty-two *RCC1* genes with significant sequence variations existed in *A. annua* genome and showed different expression patterns in different conditions, which indicated their divergent roles in response to the external environment. Though divergence existed among individual *RCC1* genes in one species, the gene number of *RCC1* genes remained conserved among eudicots. The conservation of the number of *RCC1* genes was not only observed in eudicots but also monocots [48]. Increased gene numbers of *RCC1* could be observed in genomes with recent whole genome polyploidizations [34, 49]. While, the most recent WGT of *A. annua* occurred at 58.12 Ma [50], and most of duplicated *RCC1* copies were functionally inactive with incomplete structures (Figure S2). During the re-diploidization process of post-WGD, gene deletion occurred due to dosage constraints [51]. Functional copies of *RCC1* genes were maintained over time and the *RCC1* gene loss can be an adaptive evolutionary force facing environmental challenges [45]. A negative selection was observed among *RCC1* genes as the Ka/Ks ratios were prevalently lower than 1 within or between species**,** which would eliminate deleterious mutations and maintain functional stability of *RCC1* genes [40]. As their conservation characteristics among different lineages of eudicots, studies of functional examination and regulatory mechanism deconstruction should be conducted for each *RCC1* member which could be beneficial for the whole plant research community.

*UVR8* is one of the *RCC1* family members and the well-known UV-B receptor gene [22]. UV-B radiation is an environmental stimulus, a major abiotic stress confronting living tissue. Low-dose and non-damaging UV-B regulate photomorphogenesis and metabolite biosynthesis by serving as a photomorphogenic signal [52]. The photoactivated UVR8 could transduce UV-B signal via multiple mechanisms to regulate transcription and plant growth [53]. UVR8 proteins from green algae to higher plants are functionally conserved and likely to be pivotal in mediating responses to UV-B in numerous species. Strong purifying selection pressure identified among *UVR8* orthologs in different lineages maintains its conserved function. Interestingly, the expression of *AaUVR8* showed a decreasing trend after UV-B treatment, which was different from well-known stress-tolerance genes (NAC, ERF, CBF) [54–56]. It was considered that the UV-B signal was transduced immediately by *UVR8* and relevant genes while repressors (like RUP1 and RUP2) had negative feedback regulation and repressed *UVR8* expression.

## Conclusions

In this study, a comprehensive bioinformatic analysis was conducted on the *AaUVR8* and *RCC1* gene family of *A. annua*, which would help explain the role of light signal recognition and transduction in *A. annua.* Besides, the study contributed to screening varieties with high resistance to light stress in molecular-assisted breeding.

## Materials and methods

### Identification, phylogenetic and conserved domain analysis of the *RCC1* genes in *A. annua*

Four haplotype genomes (LQ-9 haplotype 0, LQ-9 haplotype 1, HAN1 haplotype 0 and HAN1 haplotype 1) and transcriptomes from different tissues of *A. annua* were used in this study, The data were downloaded from Global Pharmacopoeia Genome Database (GPGD, http://www.gpgenome.com/) [50, 57]. The annotated AtUVR8 protein sequences were obtained from the TAIR database (http://www.arabidopsis.org). Protein sequences from *A. annua* genome were searched against the PFAM database (Pfam 32.0) using PfamScan (evalue ≤ 1e-5; http://www.ebi.ac.uk/Tools/pfa/pfamscan). Genes with hits to *RCC1* domain (PF00415) were considered as candidate *RCC1* genes. Finally, the genes were viewed and corrected using the Apollo browser [58] followed the Wang et al*.* [59] to rule out false-positive results. According to the amino acid similarity (identity ≥ 80%), the allelic (one-to-one) relationship of *RCC1* genes among haplotype genomes was confirmed.

The phylogenetic trees of 22 AaRCC1 proteins in *A. annua* LQ-9 haplotype 0 were constructed using MEGA X [60] with 1000 bootstrap replications and both neighbor-joining and maximum likelihood models. The phylogenetic tree, gene structures and PFAM domains were plotted by ggtree package [61].

### Evolutionary analysis of UVR8 in five eudicot species

*RCC1* genes of four other species, *A. thaliana*, *C. nankingense*, *H. annuus*, and *V. vinifera* were identified using same method as used for *A. annua*. The *A. annua* RCC1 proteins were searched against candidate RCC1 proteins of other species utilizing BLASTp [62] and hits with identity ≥ 40% and coverage ≥ 60% were kept. The synteny analysis between *A. annua* and other species was performed by the Multiple Collinearity Scan toolkit (MCscan**,** Python version) [63]. The Ks values of ortholog pairs or paralog pairs in five species were calculated using KaKs_Calculator2.0 [64]. Multiple sequence alignment of ortholog proteins was performed using ClustalX method with MEGA X.

### Expression analysis based on RNA-Seq data

The raw reads generated by different tissues (http://www.gpgenome.com/species/92) and corresponding transcriptome data with different treatments in A*. annua* (Table S4) deposited in PRJNA435470 (SRP133983) [65] and PRJNA601869 [17] of the NCBI were quality controlled using Skewer [66]. High-quality reads were mapped to the LQ-9 haplotype 0 genome sequences using HISAT2 [67]. The expression level of each gene was calculated with StringTie [68]**.** Differential expression of *RCC1* genes in four tissues (root, stem, leaf, and flower) and different treatments were analyzed with DESeq2 [69]. Hierarchical clustering analysis and expression level of TPM **(**transcript per million**)** values was performed using the 'pheatmap**’** package (https://cran.rproject.org/web/packages/pheatmap/) in R.

### RNA extraction and Expression analysis by quantitative PCR

The qPCR samples of *A. annua* were collected in different provinces of China (Table S3), which were identified by Li Xiang and preserved in an accessible herbarium of Artemisinin Research Center, Institute of Chinese Materia Medica, China Academy of Chinese Medical Sciences. Total RNA was extracted according to the instruction manual of the Plant Total RNA Isolation Kit (Vazyme, Nanjing, China). First-strand cDNA was synthesized with a HiScript III 1st Strand cDNA Synthesis Kit (+ gDNA wiper) (Vazyme, Nanjing, China) according to the manufacturer’s instructions. *AaActin* was used as a reference. Primers for *AaUVR8* and *AaActin* (Table S2) were designed and synthesized by Sangon Biotech Co., Ltd (Shanghai, China). The qPCR reaction was performed using the Applied Biosystems ABI 7500 PCR System (ABI, United States). The PCR amplification mixture contained 2 μl of cDNA, 10 μl of ChamQ Universal SYBR qPCR Master Mix (Vazyme Biotech Co., Ltd), 0.4 μl of 10 μM forward and reverse primers, and 7.2 μl ddH_2_O. The PCR reaction was performed with the initial denaturation step for 30 s at 95 °C; 40 cycles of 10 s at 95 °C and annealing at 60 °C for 30 s. The melting curves (60–95 °C) were used to check the specificity of each qPCR reaction. The standard curves were generated using a twofold dilution gradient of the cDNA. Amplification efficiencies (E = 10^–1/slope^-1) and correlation coefficient (R^2^) values were calculated by standard curves. The relative gene expression was calculated with the 2^–ΔΔCt^ method [70].

### Supplementary Information

**Additional file 1: Supplemental Table S1. **The alleles of *AaRCC1* in four haplotype genomes. **Supplemental Table S2.** Primers used in qPCR of *AaUVR8* and *AaActin*. **Supplemental Table 3. **qPCR samples of *A. annua*. **Supplemental Table 4.** Different treatments in *A. annua.*

**Additional file 2: Fig. S1. **The phylogenetic relationships of RCC1 family proteins in 5 species.

**Additional file 3: Fig. S2. **The duplication debris of *AaRCC1_07* and *AaUVR8* in *A. annua* genome.

**Additional file 4: Fig. S3. **The relative expression of *AaUVR8* in 18 *A. annua* samples

**Additional file 5: Fig. S4. **The expression profile of *AtRCC1* genes in UV-B treatment.

## Data Availability

The identified *RCC1* genes were deposited in the Global Pharmacopoeia Genome Database at http://www.gpgenome.com/species/92. Data supporting the findings of this work are available within the paper and its Supplementary Information files. The datasets generated and analyzed during the study are available from the corresponding author upon reasonable request.
