# Supplementary figures and images for: Genome-wide characterization of regulator of chromosome condensation 1 (RCC1) gene family in Artemisia annua L. revealed a conservation evolutionary pattern
Source: BMC Genomics. 2023 Nov 18;24:692. doi: 10.1186/s12864-023-09786-4 (PMC10657572; doi:10.1186/s12864-023-09786-4)

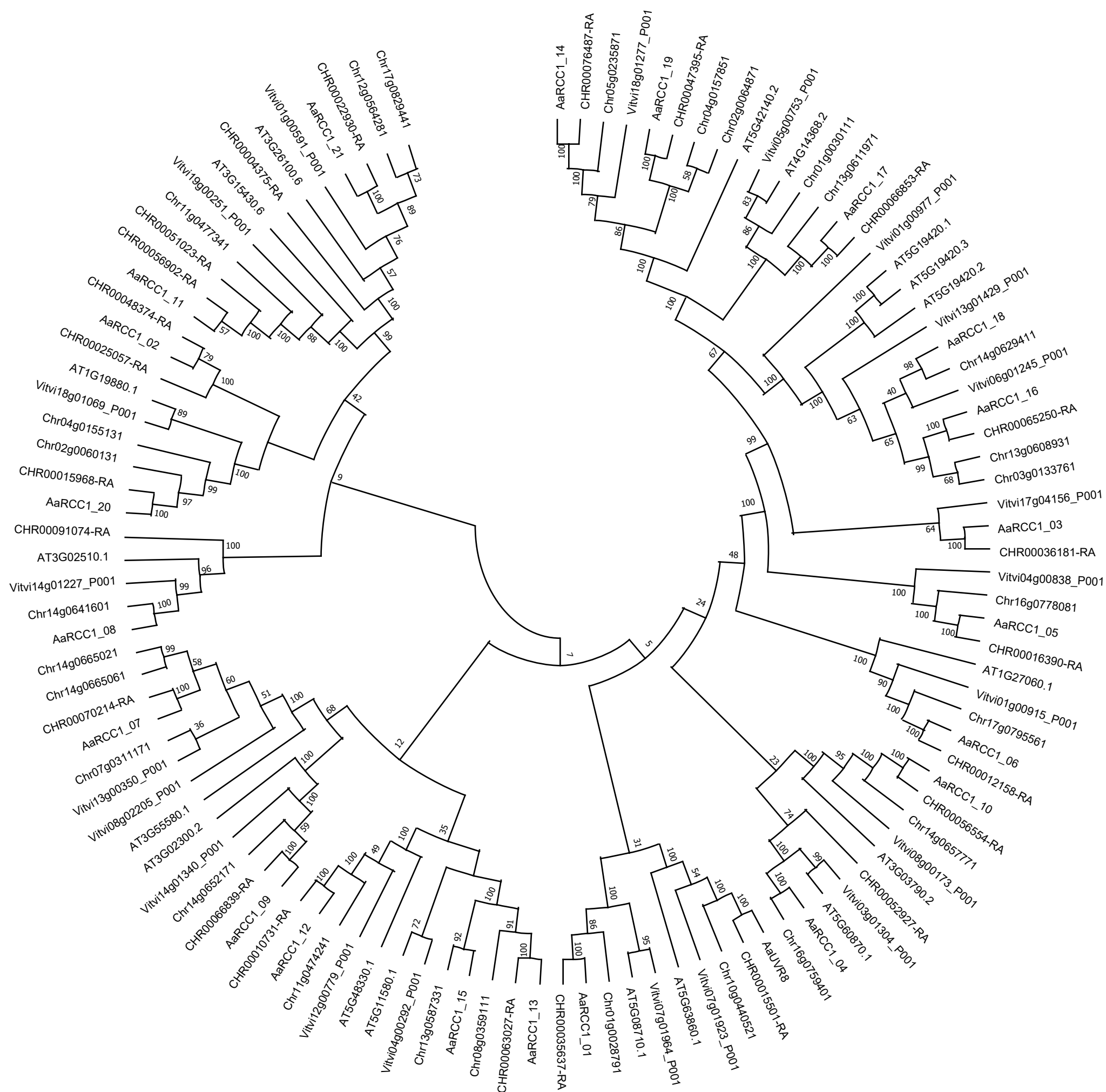

Fig. S1 The phylogenetic relationships of RCC1 family proteins in 5 species

Supplement: Supplementary file 2 — Additional file 2: Fig. S1. The phylogenetic relationships of RCC1 family proteins in 5 species. [file 12864_2023_9786_MOESM2_ESM.pdf]

A

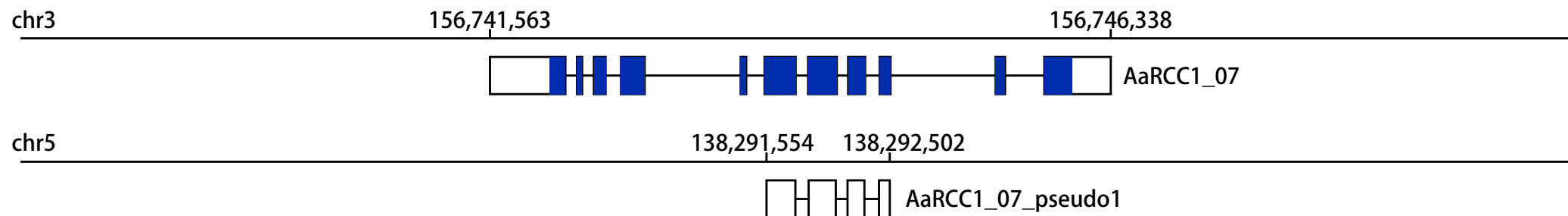

B

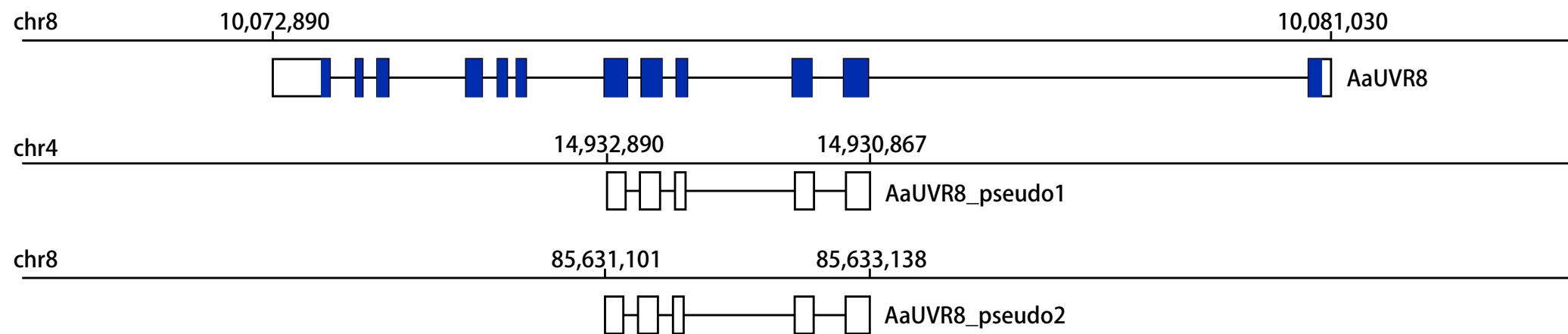

Fig. S2 The duplication debris of *AaRCC1\_07* and *AaUVR8* in *A. annua* genome

Supplement: Supplementary file 3 — Additional file 3: Fig. S2. The duplication debris of AaRCC1_07 and AaUVR8 in A. annua genome. [file 12864_2023_9786_MOESM3_ESM.pdf]

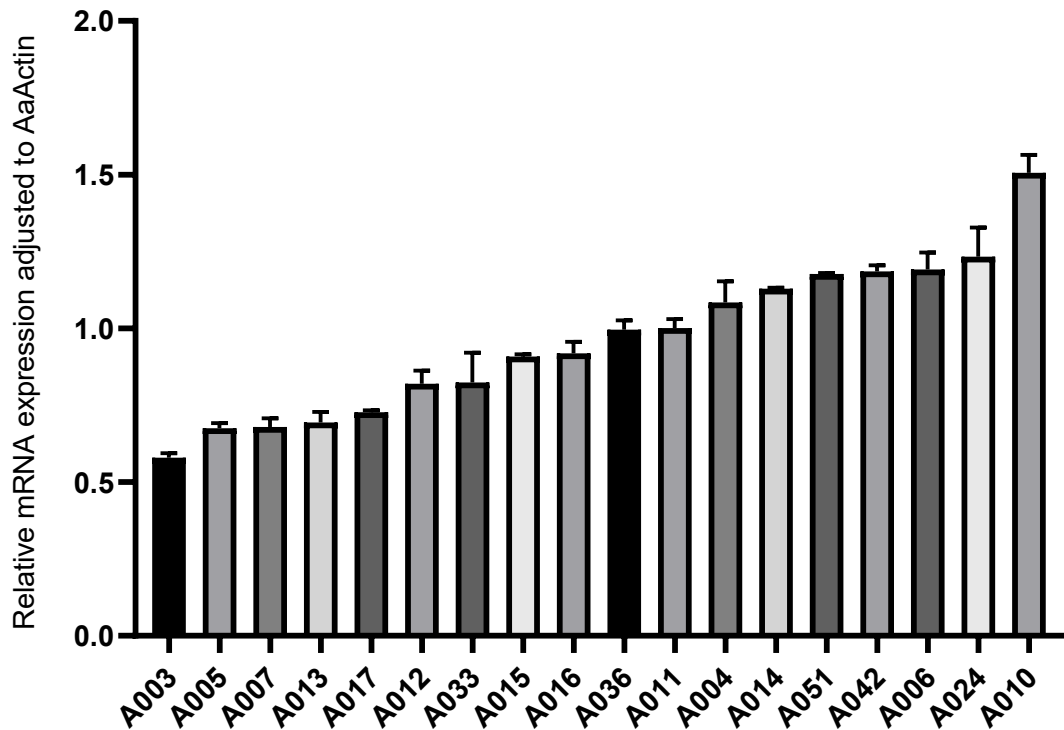

Fig. S3 The relative expression of *AaUVR8* in 18 *A. annua* samples

Supplement: Supplementary file 4 — Additional file 4: Fig. S3. The relative expression of AaUVR8 in 18 A. annua samples [file 12864_2023_9786_MOESM4_ESM.pdf]

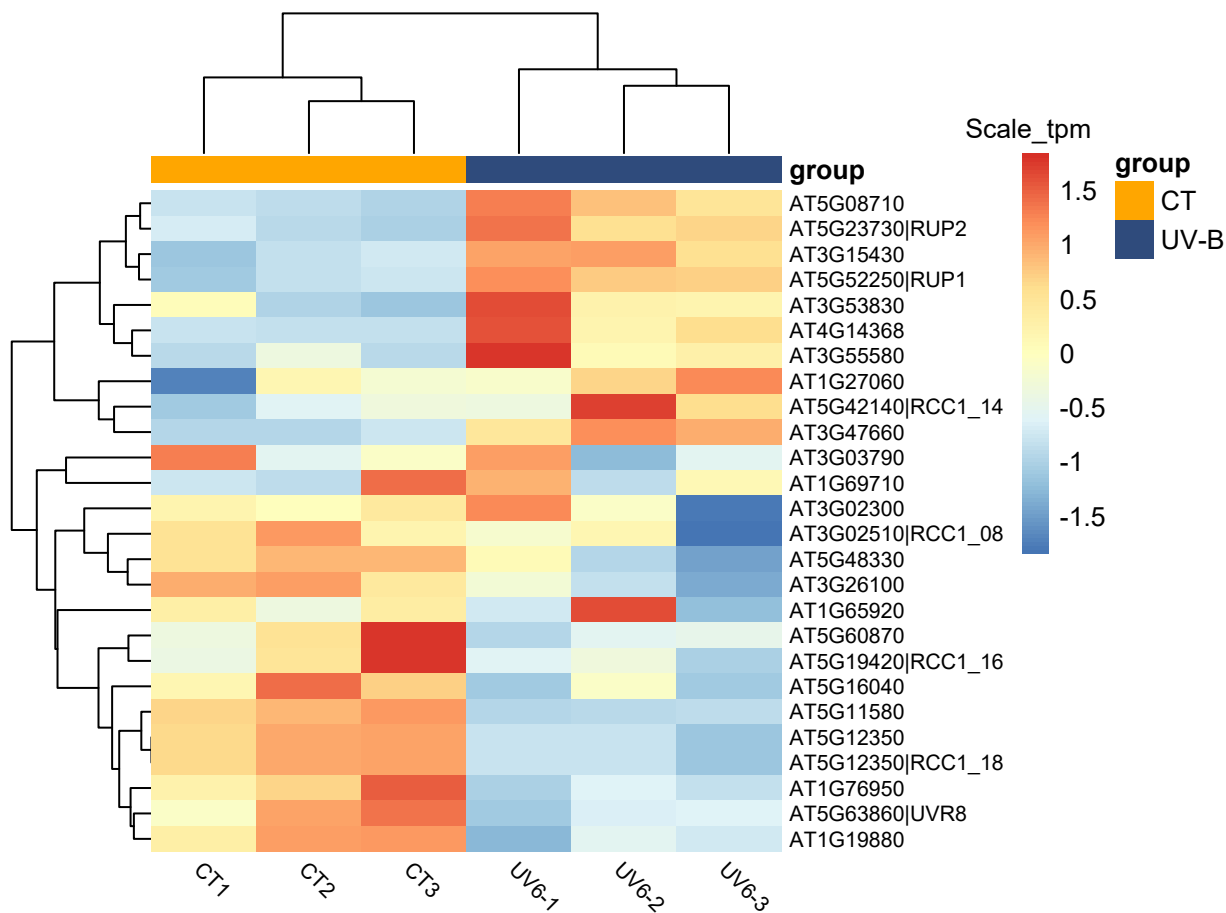

Fig. S4 The expression profile of *AtRCC1* genes in UV-B treatment

Supplement: Supplementary file 5 — Additional file 5: Fig. S4. The expression profile of AtRCC1 genes in UV-B treatment. [file 12864_2023_9786_MOESM5_ESM.pdf]
